# River temperature drives salmon survivorship: is it determined prior to ocean entry?

**DOI:** 10.1098/rsos.140312

**Published:** 2015-01-28

**Authors:** Kentaro Morita, Nakashima Ayumi, Motohiro Kikuchi

**Affiliations:** 1 Hokkaido National Fisheries Research Institute, Fisheries Research Agency, 2-2 Nakanoshima, Toyohira-ku, Sapporo, Japan; 2 Chitose Salmon Aquarium, Salmon Park Chitose, 312, Hanazono 2-chome, Chitose, Hokkaido, Japan

**Keywords:** population dynamics, mortality, salmon, river temperature

## Abstract

Early life is believed to be a critical stage for determining survivorship in all fish. Many studies have suggested that environmental conditions in the ocean determine the fry-to-adult survival rate of Pacific salmon but few investigations have been conducted on the importance of the brief freshwater period during the seaward migration on overall survivorship. Here, we found that most of the variation in survivorship of hatchery-reared chum salmon (*Oncorhynchus keta*) was explained by river temperature during the fry stage, despite spending most of their life (approx. 99%) at sea. After the annual release of a constant number of fry, the number of fry moving through the river at a downstream location varied greatly. The number of returning adults was positively correlated with the number of fry moving downstream. This result suggests that most salmon mortality occurred prior to ocean entry, and that short-term mortality in the river is a key factor determining major fluctuations in total mortality. Although marine mortality is often invoked in the literature as a key factor determining total mortality of chum salmon, attention should also be paid to freshwater mortality to understand the population dynamics of this species.

## 2. Introduction

Determinants of animal mortality over time are of central concern in ecology and wildlife management. A ‘key factor’ is defined as a component of a life table that is responsible for major fluctuations in overall survivorship and therefore can be used to predict population trends [1]. Considerable evidence in marine fish indicates that high mortality occurs during early life [2]. Hence, this could be considered a key factor. Early ocean life has also been suggested as a critical life stage to determine survivorship (fry-to-adult survival rate) of Pacific salmon [3–5]. Many studies have shown a covariation between Pacific salmon survivorship and oceanographic conditions (e.g. sea surface temperature) [3–5]. However, Pacific salmon are anadromous fish and spawn in freshwater. Juveniles spend days to years in their freshwater rearing habitat before moving to sea. Although most of their life is spent at sea, their brief freshwater life during the seaward migration should not be ruled out as a determinant of overall survivorship.

Several studies have suggested that riverine environmental conditions, such as temperature and discharge, significantly affect freshwater mortality of salmonid fish prior to ocean entry [6,7]. However, few attempts have been made to relate riverine environmental conditions with overall Pacific salmon survivorship.

Here, we asked whether the brief freshwater life-history stage during the seaward migration is important to determine hatchery-reared chum salmon (*Oncorhynchus keta*) survivorship, which migrate to sea 10–30 days after release [8,9] and spend 3–6 years at sea. Because temperature is one of the most important environmental factors affecting physiological processes in ectotherms, we hypothesized that river temperatures during out-migration would affect salmon fry survival. Hence, we examined the relationships among river temperatures, the surviving number of fry moving through the river and overall survivorship of chum salmon.

## 3. Material and methods

**Figure 1.**
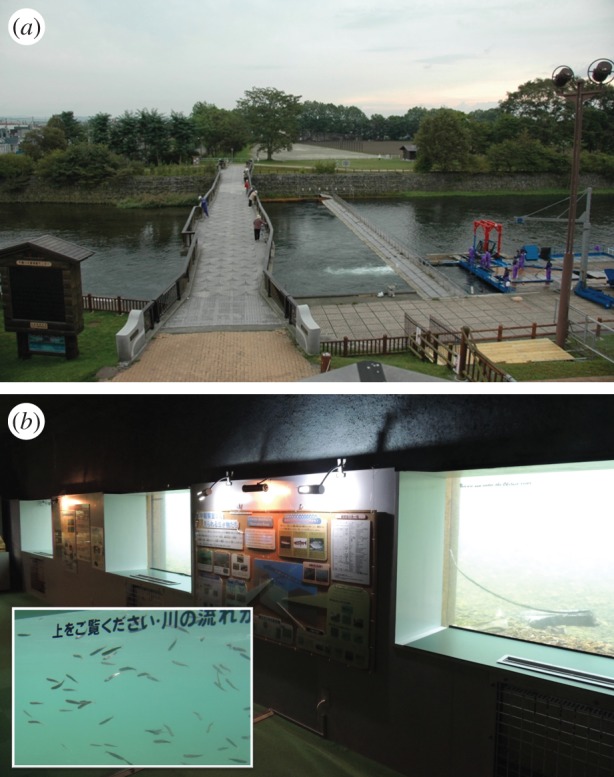
(*a*) The weir located in the Chitose River. (*b*) The CSA observation windows. The inset illustrates chum salmon fry moving past the window in April 2008 (http://www.city.chitose.hokkaido.jp/tourist/salmon/e-html/observ.html).

The relationship between river temperature and salmon survivorship was examined in the Chitose River, Japan, where 30 million chum salmon fry are released annually from the Chitose Field Station (CFS) of the Hokkaido National Fisheries Research Institute. The distance from the river mouth to the CFS is approximately 80 km. Almost all fry released have thermally induced otolith marks [10] for each brood year since 2001 [11]. Returning adults were captured each year between August and December at a weir located 10 km downstream from the CFS (figure 1*a*). In addition to daily catches, approximately 100 adults (approx. 50 males and 50 females) were sampled every 10 days; age was determined from scales [12], and they were confirmed to be hatchery or wild fish by checking their otolith marks. Then, survivorship (fry-to-adult survival) of chum salmon fry (*φ*_*t*_) was calculated for each brood year, *t*, as

$$\varphi_{t}=\frac{\left(\sum_{i}\sum_{j}p_{Mi,j,t+i}\cdot y_{Mj,t+i}+\sum_{i}\sum_{j}p_{Fi,j,t+i}\cdot y_{Fj,t+i}\right)}{x_{t}},$$

where *p*_*Mi*,*j*,*t*_ and *p*_*Fi*,*j*,*t*_ is the proportion of otolith-marked fish of age *i* and season *j* in year *t* for males and females, respectively, *y*_*Mj*,*t*_ and *y*_*Fj*,*t*_ is the number of male and female fish caught in season *j* in year *t*, and *x*_*t*_ is the number of otolith-marked fry released in brood year *t*. The 2001–2008 brood years were targeted in this study because most chum salmon had returned by age 5 years (i.e. adult samples were included up to December 2013).

Daily river temperatures and the number of fry moving past the Chitose Salmon Aquarium (CSA), located 10 km downstream from the fry release site, were measured. Fish were counted every morning around 8.30 from in-river observation windows (1×2 m rectangle, *n*=7) by CSA staff (figure 1*b*). The number of salmon fry counted was used as an abundance index for fry moving downstream. Water transparency, which could affect visibility, was not considered, but it was usually adequate on most days. Because the salmon fry included unmarked wild fish, the abundance index was multiplied by the fraction of hatchery fish estimated previously [11]. Daily river discharge data at Nishikoshi, located 2 km downstream from the CSA, were obtained from the Water Information System provided by the Ministry of Land, Infrastructure, Transport and Tourism (http://www1.river.go.jp/). The distances from the river mouth to the CSA and CFS were 70 and 80 km, respectively.

## 4. Results

**Figure 2.**
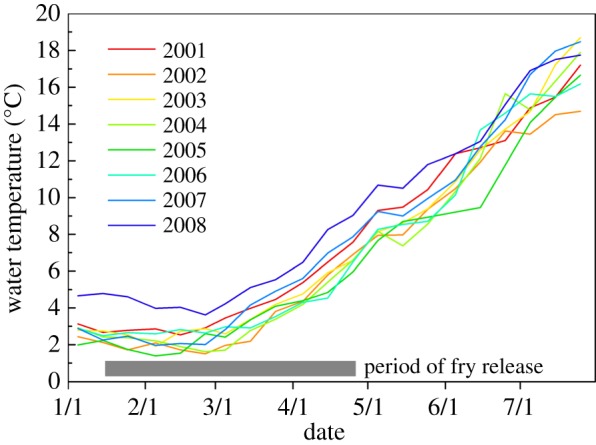
Seasonal changes in water temperature in the Chitose River for successive 10-day periods during out-migration of chum salmon fry of the 2001–2008 brood years.

**Figure 3.**
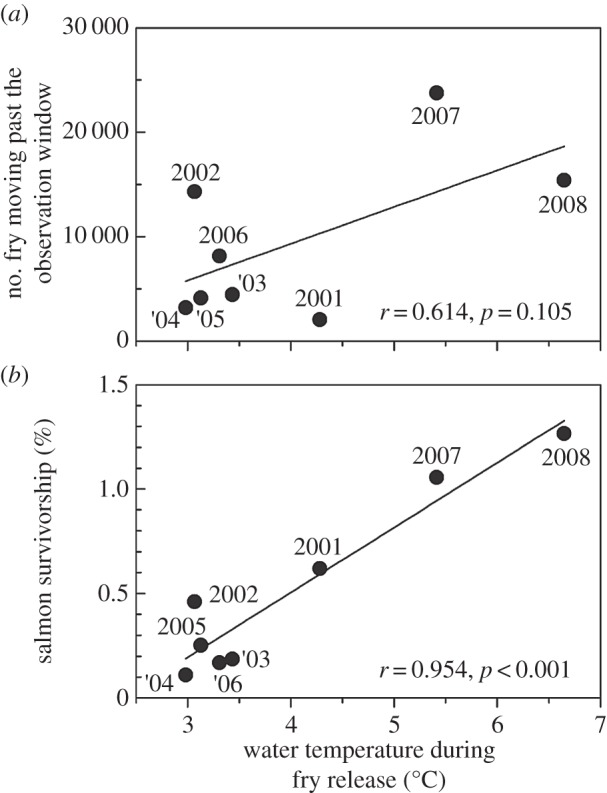
(*a*) Relationship between average water temperature during the period of fry release and the number of chum salmon fry moving past the observation windows. (*b*) Relationship between average water temperature during the period of chum salmon fry release and survivorship (fry-to-adult survival rate).

**Figure 4.**
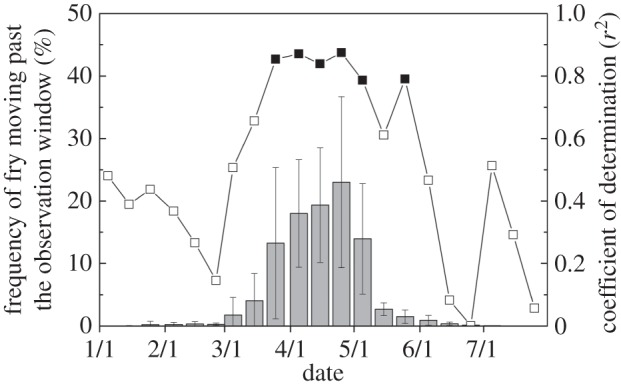
Coincidence between average frequency ± s.d. of chum salmon fry moving past the observation windows during each 10-day period (left axis; grey bars) and the coefficients of determination between survivorship and water temperature for each 10-day period (right axis; black squares, *p*<0.01; white squares, *p*>0.01).

**Figure 5.**
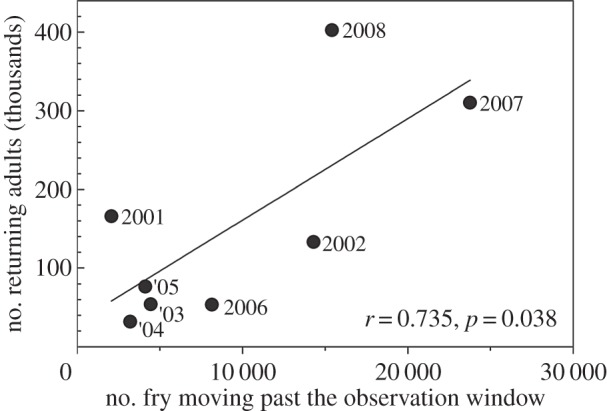
Relationship between the number of chum salmon fry moving past the observation windows and the number of returning adults by brood year.

River temperature increased from 2°C to 10°C during the salmon fry out-migration period and significant year-to-year variation was observed (figure 2). The period of fry release varied from mid-January to late April, but the average fry release date varied little between years (late March to early April). The number of fry passing the in-river observation windows tended to be positively correlated with the average river temperature at fry release, but it was not statistically significant (figure 3*a*). Released fry survivorship was significantly correlated with average river temperature during fry release (figure 3*b*). The salmon fry passed the in-river observation windows from January to July with peak passage between March and May, which was consistent with the significant correlation between survivorship and seasonal river temperatures (figure 4). Although the same number of fry was released each year, the number of fry moving past the observation windows fluctuated greatly. An approximate 12-fold year-to-year variation was observed in the number of fry moving past the observation windows and in the number of returning adults. The number of returning adults was positively correlated with the number of fry moving past the observation windows (figure 5).

Survivorship of released fry was not significantly correlated with the average date of fry release (*r*=0.489, *p*=0.219) but was significantly correlated with average river discharge during fry release (*r*=−0.724, *p*=0.042). The effects of the date of fry release and river discharge on survivorship were not significant after incorporating the effect of river temperature (multiple regression: release date, *p*=0.765; discharge, *p*=0.376; temperature, *p*=0.008). Released fry survivorship was negatively correlated with the proportion of fry released between January and February (*r*=−0.763, *p*=0.028), but it was not significant after incorporating the effect of river temperature (multiple regression: *p*=0.903). Only river temperature was a significant explanatory variable for the annual variation in survivorship of released fry.

## 5. Discussion

Most of the variation in chum salmon survivorship in the Chitose River was explained by river temperature during the fry stage, despite that chum salmon spend the majority of their life (approx. 99%) at sea. Most fry released into the Chitose River migrate to sea within 10 days [8] and spend 3–6 years in that environment. Although the same number of fry was released each year, the abundance index of fry moving past the river observation windows and the number of returning adults varied about 12-fold. Moreover, the number of returning adults was positively correlated with the number of fry moving downstream. These findings indicate that a large proportion of salmon mortality occurred prior to ocean entry, and that short-term mortality in the river is a key factor determining major fluctuations in overall survivorship. Our demonstration of spring temperature-driven salmon survivorship represents a lagged effect of climate because chum salmon spend 3–6 years at sea before they return to their natal river.

The relationship between river temperature and overall survivorship was stronger than the relationship between river temperature and the abundance index for fry moving downstream. One possible reason is that the abundance index of fry moving downstream is less accurate than counts of returning adults, so the observation uncertainty leads to a weaker relationship with the fry abundance index. Another possibility is that there is a delayed effect of river temperature on survival. Fry must migrate an additional 70 km prior to ocean entry from the fry counting site, so freshwater mortality would be ongoing at the fry counting site. Some studies have suggested that delayed mortality occurs in relation to earlier hydrosystem experience during the downstream migration of Pacific salmon [13,14].

Several mechanisms explain why a lower river temperature in spring leads to poorer survivorship. We derived three possible explanations. First, a temporal mismatch may be occurring with food resources. Salmon fry feed on aquatic insects, such as chironomids [15], whose availability and digestion are limited by low temperature. Second, a low tolerance for low temperatures may result in high mortality. Both hatchery and wild chum salmon are incubated in upstream spring-fed waters at a relatively constant temperature (6–11°C) [16,17]. However, fry encounter very low temperatures (2–5°C) during their downstream migration, particularly early in spring [17]. An experimental study demonstrated the tolerance to low temperatures by young Pacific salmon [18]. The lethal temperature for chum salmon fry acclimated at 10–15°C was 0.5–4.7°C [18]. Therefore, the observed range of ambient temperatures in early spring could cause thermal damage to salmon fry. Third, a slower swimming speed at low temperature may increase vulnerability to predation (e.g. birds); several studies have shown that swimming speed decreases at low temperatures [19,20]. These three explanations are not mutually exclusive, and all might contribute to lower survival in freshwater during the seaward migration at low temperatures.

Hatchery programmes involving mass release of artificially propagated fish have been implemented to supplement wild populations and increase the fishery harvest. However, hatchery fish usually have a fixed release time despite the significant year-to-year variation in river temperature at the time of release. Moreover, downstream out-migration of hatchery fish occurs about one month earlier than that of wild fish in the river we studied [21]. Therefore, hatchery fish may be more vulnerable to the impact of low river temperature during the seaward migration early in spring. Releasing date-specific otolith-marked fish showed that high survivorship (more than 1%) occurs only for marked groups released in and after late March in the Chitose River [22]. River temperature could serve as an indicator for appropriate timing of fry release. However, there are other considerations involved in the decisions to release, such as hatchery capacity limitations (i.e. keeping a large number of growing fry in good health is difficult) and the agricultural use of river water after the growing season (i.e. salmon fry might stray into agricultural water).

Recent studies have confirmed high mortality of Pacific salmon during the first month of migration in freshwater [17,23–25]. For example, the estimated survival rate of marked chum salmon fry during a downstream migration over 11.6±4.4 days was 16.5% [17]. Approximately 60–74% of hatchery steelhead trout (*Oncorhynchus mykiss*) smolts and 16–29% of wild smolts died prior to ocean entry [25]. A review and meta-analysis of Pacific salmon survival rates showed that the inter-annual variance in mortality is greater in freshwater than in marine habitats, suggesting that the freshwater period is important, even for pink and chum salmon that spend most of their life at sea [26]. In addition, river temperature also affects sockeye salmon (*Oncorhynchus nerka*) adult survival during their upstream migration [27,28]. Although marine mortality is often used as a key factor determining overall chum salmon survivorship in the literature, attention should be paid to freshwater mortality when considering chum salmon population dynamics.

There is growing recognition of the impact of climate warming on salmonid fish populations [29,30]. Climate warming is usually suggested as a negative force decreasing Pacific salmon survivorship. However, our study found a positive association between spring temperature and survivorship of hatchery-reared chum salmon. Because spring air temperature has shown an increasing trend over the last century [31], recent climate warming could have positively affected the survival rate of hatchery-reared chum salmon in recent decades. However, it is uncertain whether further climate warming will enhance hatchery-reared chum salmon survivorship.

## Supplementary Material

The data used in this paper

## Data accessibility

The datasets supporting this article are uploaded as electronic supplementary material.
