# Supplementary material for: River temperature drives salmon survivorship: is it determined prior to ocean entry?
Source: R Soc Open Sci. 2015 Jan 28;2(1):140312. doi: 10.1098/rsos.140312 (PMC4448787; doi:10.1098/rsos.140312)
Supplement: The data used in this paper [file rsos140312supp1.pdf]

Brood year	2001	2002	2003	2004	2005	2006	2007	2008
Fry migration year	2002	2003	2004	2005	2006	2007	2008	2009
Number of fry released (thousands)	27097	30121	28975	29015	30265	31718	31149	31790
Number of otolith-marked fry released (thousands)	26766	28870	28975	29015	30265	31718	29386	31790
Proportion of marked fry	0.988	0.958	1.000	1.000	1.000	1.000	0.943	1.000
Number of otolith-marked returning adults	165927	133105	54086	31988	76449	53460	310264	402545
Survivorship (%)	0.620	0.461	0.187	0.110	0.253	0.169	1.056	1.266
Number of salmon fry counted	2490	15428	5649	4521	4709	9101	26782	15728
Fraction of hatchery fish	0.840	0.967	0.788	0.710	0.875	0.897	0.941	0.980
Estimated number of otolith-marked fry	2066	14300	4451	3211	4119	8161	23764	15415
Water temperature during release	4.28	3.07	3.43	2.98	3.13	3.31	5.41	6.65
Discharge during release (m3/s)	16.24	16.34	18.12	15.81	18.29	21.98	13.86	14.26
Average date of fry release	2002/4/7	2003/4/8	2004/4/4	2005/4/7	2006/3/24	2007/3/21	2008/4/6	2009/4/8
Start date	2002/2/6	2003/2/7	2004/1/16	2005/2/5	2006/1/31	2007/2/1	2008/3/14	2009/3/19
End date	2002/4/26	2003/4/21	2004/4/21	2005/4/20	2006/4/20	2007/4/20	2008/4/18	2009/4/20
Temperature in early-Jan	3.13	2.43	2.79	2.91	1.99	2.89	2.88	4.67
Temperature in mid-Jan	2.67	2.12	2.75	2.42	2.23	2.49	2.26	4.79
Temperature in late-Jan	2.78	1.72	2.54	2.38	1.74	2.66	2.46	4.6
Temperature in early-Feb	2.86	2.1	1.92	2.21	1.39	2.59	1.97	3.98
Temperature in mid-Feb	2.54	1.72	2.72	1.9	1.54	2.83	2.06	4.04
Temperature in late-Feb	2.91	1.5	2.88	1.61	2.6	2.64	2.01	3.63
Temperature in early-Mar	3.46	1.96	2.59	1.71	2.41	2.97	2.83	4.23
Temperature in mid-Mar	3.98	2.19	3.37	2.78	3.35	2.91	4.16	5.11
Temperature in late-Mar	4.45	3.81	4.19	3.39	4.09	3.53	4.91	5.54
Temperature in early-Apr	5.38	4.36	4.76	4.19	4.39	4.3	5.6	6.47
Temperature in mid-Apr	6.49	5.79	5.91	5.4	4.84	4.54	6.98	8.25
Temperature in late-Apr	7.58	6.9	6.58	6.6	5.96	6.5	7.86	9.04
Temperature in early-May	9.31	7.94	8.14	8.15	7.66	8.27	9.25	10.7
Temperature in mid-May	9.49	7.96	8.62	7.37	8.7	8.54	9	10.5
Temperature in late-May	10.4	9.37	9.4	8.57	8.94	8.72	9.95	11.8
Temperature in early-Jun	12.4	10.5	10.9	10.3	9.22	10.1	11	12.4
Temperature in mid-Jun	12.7	11.9	12.6	12.1	9.46	13.7	12.8	13.1
Temperature in late-Jun	13.1	13.6	13.7	15.7		14.6	14.2	15.1
Temperature in early-Jul	14.9	13.4	14.7	14.8	14.1	15.6	16.7	16.9
Temperature in mid-Jul	15.5	14.5	17.2	16.3	15.5	15.5	18	17.5
Temperature in late-Jul	17.2	14.7	18.7	17.9	16.6	16.2	18.5	17.7
Proportion of fry released in Jan	0.000	0.000	0.024	0.000	0.000	0.000	0.000	0.000
Proportion of fry released in Feb	0.077	0.111	0.085	0.041	0.104	0.091	0.000	0.000
Proportion of fry released in Mar	0.218	0.076	0.274	0.198	0.635	0.607	0.288	0.306
Proportion of fry released in Apr	0.704	0.814	0.617	0.761	0.260	0.301	0.712	0.694
